# The exploratory dataset of isotopic composition of different water sources across Kazakhstan

**DOI:** 10.1016/j.dib.2024.110360

**Published:** 2024-03-22

**Authors:** Vadim Yapiyev, Nurlan Ongdas, Sylvia Pinkerneil, Kanat Samarkhanov, Arman Kabdeshev, Yergali Karakulov, Murat Muzdybaev, Aksholpan Atalikhova, Catalin Stefan, Jay Sagin, Milovan Fustic

**Affiliations:** a International Science Complex Astana, Kabanbay Batyr Ave 8, Astana 020000, Kazakhstan; b School of Mining and Geosciences, Nazarbayev University, 53 Kabanbay Batyr Ave, Astana, 010000, Kazakhstan; c The Environment & Resource Efficiency Cluster (EREC), Nazarbayev University, 53 Kabanbay Batyr Ave, Astana, 010000, Kazakhstan; d Research Group INOWAS, Department of Hydro Sciences, Technische Universität Dresden, 01069 Dresden, Germany; e Section Climate Dynamics and Landscape Evolution, Helmholtz Centre Potsdam, GFZ German Research Centre for Geosciences, Telegrafenberg, 14473 Potsdam, Germany; f Kazakh-British Technical University, Tole Bi Street 59, Almaty 050000, Kazakhstan; g Western Michigan University, Kalamazoo, 49008, MI, United States; h University of Calgary, Department of Earth, Energy, and Environment, 2500 University Drive NW, Calgary, AB T2N 1N4, Canada

**Keywords:** Stable isotopes, Isotope hydrology, Oxygen-18, Deuterium, Local meteoric water line, Precipitation, Lake, Central Asia

## Abstract

This work presents the dataset of stable water isotopes of oxygen and hydrogen measured in water samples from different sources (precipitation, surface water, groundwater, tap water) across Kazakhstan from 2017 to 2018 and from 2020 to 2023. The dataset includes results on isotopic composition of 399 water samples, namely precipitation: event-based (*n* = 108), cumulative monthly (*n* = 22); surface water: lakes, reservoirs, brooks, rivers, channels (*n* = 175), groundwater: shallow and artesian groundwater, spring (*n* = 85), tapwater (*n* = 9). For each sample name of the source, location, latitude, longitude and date of sampling, measurement uncertainty (one standard deviation) are available. The samples were assessed by plotting the data in dual δ^18^O vs. δ^2^H isotope space with reference to values found in the published literature and fitting a linear regression equation for Astana (event) precipitation. Overall, this is the first dataset covering wide range of sources across Kazakhstan, which could be used by global and regional water resource assessments and studies such as tracing water sources, hydrograph separation and end-member analyses, isotope mass balance, evapotranspiration partitioning, residence time analysis and groundwater recharge.

Specifications Table

**Table:** 

Subject	Hydrology and Water quality
Specific subject area	Baseline water stable isotope dataset for use in isotope hydrology, hydrogeology, and watershed studies across Kazakhstan
Data format	*Stable water isotope ratios of oxygen and hydrogen in per mil (‰) normalized to Vienna Standard Mean Ocean Water (VSMOW2) with measurement uncertainties (1 standard deviation)*
Type of data	Tables, figure, MS Excel file
Data collection	Rainfall event samples were collected during abundant precipitation events using a large plastic container and immediately transferred into the vials and sealed. Monthly cumulative rain samples were collected by Palmex Rain Sampler (RS-1, Palmex d.o.o) and at the end of the month or the first day of the next month samples were transferred into the sealed bottles. Snow samples were collected after the end of the snowfall event and melted at room temperature before filling in 20 mL borosilicate glass or plastic scintillation vials with screw caps. Lake water and streamflow samples were collected by grab sampling at the shoreline during field trips. Groundwater samples were collected from boreholes using a bailer and transferred to the sealed bottles. Lake water, groundwater and streamflow samples were usually collected in duplicate during each sampling. Immediately after collection, all samples were sealed with Parafilm M to avoid evaporation. The samples were stored at room temperature or in a refrigerator until analysis. Samples were analyzed using two methods (Instruments): (1) Samples collected in 2017–2018 were analysed at GFZ German Research Centre for Geosciences, Potsdam, on Picarro water isotope analyzer (model L2130-i, Picarro, Inc), see *Laboratory analysis* section in Methods. (2) Samples collected from 2020 to 2023 were analysed at Nazarbayev University on Liquid Water Isotope Analyser (LGR IWA-912, ABB Ltd.) see *Laboratory analysis* section in Methods.
Data source location	Kazakhstan-wide, Astana, one lake sample from Kyrgyzstan
Data accessibility	Repository name: Mendeley Data Data identification number: https://data.mendeley.com/datasets/2fcyxgk77f/1

## 1 Value of the Data

• This is the first dataset [1] on isotopic composition of different water sources across Kazakhstan, that can be a useful reference for further isotope studies of Kazakhstan's water resources.
• The dataset contains the first three-year data of event-based (from the samples collected during or immediately after precipitation events, *n* = 87) and monthly precipitation data (*n* = 22) for Northern Kazakhstan (Astana city) that allows to derive Local Meteoric Water Line for this region (Fig. 2).
• The presented data has isotope values from: large endorheic lakes in Central Asia such as Lake Balkhash, North (Small) Aral, Tushybas lake, Issyk-Kul Lake; and smaller regional lakes e.g. Kambash lake in the Aral Sea Basin; rivers e.g. Syr Darya, Nura, Yesil, Ertis (Irtysh).
• This dataset contains data on groundwater isotope composition from both shallow (unconfined) and artesian (confined) aquifers.
• It extends the data of the previously published local dataset of precipitation, groundwater, streams and lake samples [2] on Burabay collected from 2015 to 2016 used to study isotope mass balance of lakes and regional hydrology.
• The dataset also contains measurement error (one standard deviation) that can be used for uncertainty estimation.

## 2 Background

The main goal for producing this dataset was to generate a baseline exploratory data on isotopic composition of water samples from different sources for whole Kazakhstan. Global and regional assessments of water resources and climate based on environmental tracers often rely on compilation of data from past published work [3,4] or decades of observations [5]. In many cases, such analyses are often limited to data-rich regions of North America, Europe or rely on the older data [6,7]. Moreover, in the published isotope studies in Kazakhstan [8,9] the underlying data is rarely available [2]. Considering the large area of Kazakhstan and wide range of landscapes and climate zones, the dataset can serve a good reference for global and regional groundwater recharge, isotope mass balance, paleoclimate and end-member analysis studies or aid local investigations [10].

## 3 Data Description

**Fig. 1:**
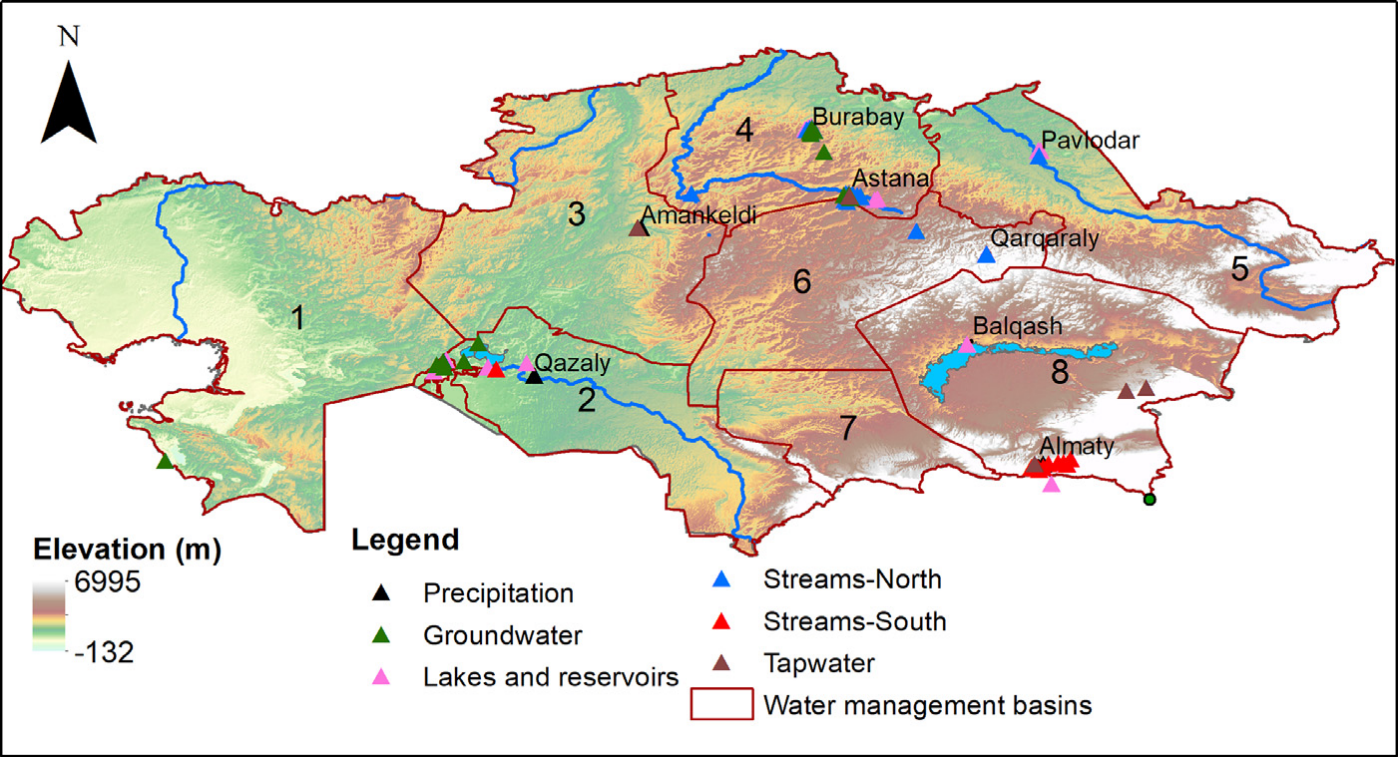
Map of Kazakhstan with the sample collection locations. The Kazakh water management basins: 1) Zhaiyk-Caspian, 2) Aral-Syr Darya, 3) Tobyl-Torgai, 4) Yesil, 5) Ertis, 6) Nura-Sarysu, 7) Shu-Talas, 8) Balkhash-Alakol.

**Fig. 2:**
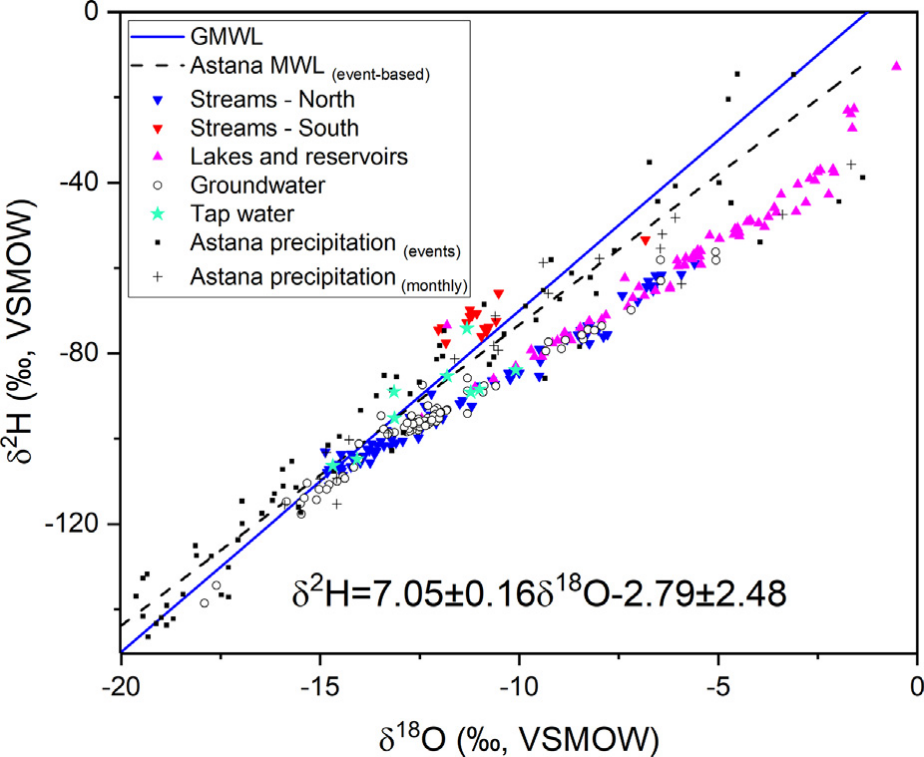
The biplot of oxygen-hydrogen isotope composition (precipitation for Astana, surface water, groundwater, tap water). Astana Meteoric Water Line (MWL) is based on ordinary linear regression (δ^2^H=7.05±0.16δ^18^O-2.79±2.48, R^2^=0.96) of event-based precipitation samples (*n* = 87) collected from 2021 to 2023. Global Meteoric Water (GMWL) is given as a global reference [11]. Streams (rivers, brooks, channels) are visually separated into North (blue inverted triangle) and South (red inverted triangle) part of Kazakhstan.

The presented dataset contains results on stable isotopic characterization of water samples collected during 2017–2018 and 2020–2023 across Kazakhstan (Fig. 1), which is located in the center of Eurasia. The data attached to the paper [1] is an original dataset showing the oxygen and hydrogen isotopic ratios (δ^18^O and δ^2^H) expressed in per mil (‰) after normalization of raw measurements with international accepted reference standards (i.e. VSMOW2 and SLAP2) along with measurements error reported as one standard deviation. The dataset (*n* = 399) consist of following water source types: precipitation (*n* = 130) - event precipitation (*n* = 108), monthly cumulative (*n* = 22); surface water (*n* = 175) – rivers (*n* = 69), brooks (*n* = 19), channels (*n* = 7), lakes (*n* = 78), reservoirs (*n* = 2); groundwater (*n* = 85) - shallow groundwater (*n* = 76), artesian (free-flowing) groundwater (*n* = 8), a spring (*n* = 1); tap water (*n* = 9). The Excel spreadsheet containing data has five tabs: 1) Precipitation events; 2) Precipitation monthly; 3) Surface water; 4) Groundwater; 5) Tap water. The tabs have the following columns: Sample no, Sample ID, Sample type, Sample subtype, Event type (for event precipitation), Sample description, Date, Location, Coordinate 1, (Lat N), Coordinate 2, (Lon E), Comment, Measurement method, δ^18^O, ‰, 1σ (standard deviation for oxygen), δ^2^H, ‰, 1σ (standard deviation for hydrogen). Fig. 2 illustrates Local Meteoric Water Line for Astana (event-based) with reference to Global Meteoric Water Line (GMWL) and the rest of isotope values in the dataset presented as the points in the dual (δ^18^O vs δ^2^H) isotope space.

## 4 Experimental Design, Materials and Methods

### 4.1 Field methods and sampling

The water samples were usually collected in 20 ml plastic or borosilicate glass scintillation vials with screw caps and sealed with Parafilm M to prevent evaporation. The samples were stored at room temperature at a dark place or in a refrigerator. Prior to analysis, the samples were usually filtered through 0.45 µm PTFE syringe filter in 2 ml screw top vials with PTFE caps following the established procedure [2]. Samples were labeled in the field with sample ID, date and location. The coordinates were recorded using mobile phone GPS or available GPS device and verified afterwards with Google Earth.

Rainfall event samples were collected during abundant precipitation events using a large plastic container, where the water was immediately transferred into the vials and sealed. Monthly cumulative rain samples were collected by Palmex Rain Sampler (Model RS-1, Palmex d.o.o.) located at approximately 1.5 m height at Nazarbayev University campus at the end of the month or the beginning of the next month. Snow samples were collected after the end of the snowfall event and melted at room temperature before filling into vials. Lake water and streamflow samples were collected during the ice-free period by grab sampling at the shoreline and avoiding poorly mixed zones. Groundwater samples were collected from boreholes using a bailer and transferred to sealed bottles, or from the taps for free-flowing (artesian) wells. Lake water, groundwater and streamflow samples were usually collected in duplicate during each sampling.

### 4.2 Laboratory analysis

The samples collected in 2017–2018 were analyzed at GFZ (German Research Centre for Geosciences, Potsdam, Germany) on the Picarro isotope analyzer (model L2130-*I*, see below the detailed description). Before the shipping to GFZ, the samples were filtered through 0.45 μm PTFE syringe filter into 2 ml screw-top vials with PTFE caps without headspace and sealed with Parafilm M. The samples were placed in a vial case and sealed in a polystyrene foam box to prevent freezing (the samples were shipped in the winter) and posted via a fast courier service. The samples collected in 2020–2023 were analysed at Nazarbayev University on Los Gatos Research (LGR) Liquid Water Isotope Analyser (model IWA-912, ABB, Ltd, see below the detailed description). The samples in the dataset were accordingly marked in the column ‘Measurement method’ – Picarro or LRG.

### 4.3 The Picarro method

Water stable isotope δ^18^O and δ^2^H were measured with a Wavelength Scanning Cavity Ring-Down Spectroscopy (WS-CRDS) using a water isotope analyser Picarro L2130-*i* equipped with an A0211 vaporizer and an A0325 autosampler (Picarro Inc., USA) performed at the stable isotope laboratory at the German Research Centre for Geosciences in Potsdam.

About 1.5 ml aliquots of filtered water samples were filled into glass vials and capped with silicon Teflon septa. All samples were measured within an optimized sequence layout (i.e. with independent prepared quality control samples and two internal laboratory reference standards) in high precision mode with nitrogen as carrier gas and the sample sizes were kept always at the same amount of water [12].

Raw data is then post-run corrected for analytical effects, i.e. drift and normalization to the international certified standard reference materials VSMOW2/SLAP2 scale, following the procedure described by van Geldern and Barth [12]. To reduce the memory effect, only the mean of the last 4 of 10 measurements of each sample were used for data evaluation.

The isotopic ratios are reported using the standard delta notation in per mil (‰) and the analytical precision (1σ standard deviation) is <0.05‰ for δ^18^O and <0.3‰ for δ^2^H.

### 4.4 The LGR method

The samples were analysed on Liquid Water Isotope Analyser (Model IWA-912, ABB Ltd) with PAL LSI autosampler (CTC Analytics AG, Switzerland) according to the manufacturer procedures. Manufacturer's reported analytical errors of the instrument are ≤±0.2‰ for δ^18^O and ±0.8‰ for δ^2^H. The following standards were used: primary, supplied by International Atomic Energy Agency (IAEA) Standard Mean Ocean Water (VSMOW2) and Standard Light Antarctic Precipitation (SLAP2); the manufacturer (LGR) supplied 1E: δ^18^O = −21.28‰, δ^2^H= −165.7‰, 2E: δ^18^O = −16.71‰, δ^2^H = −123.8‰, 3E: δ^18^O = −11.04‰, δ^2^H = −79.6‰:, 4E: δ^18^O = −7.81‰, δ^2^H = −49.2‰ with uncertainties ±0.15‰ and ±0.5‰ for oxygen and hydrogen; and in-house standards calibrated against IAEA and LGR: Burabay Lake Water (BLW) δ^18^O = −6.49 ± 0.14‰, δ^2^H = −69.2 ± 0.6‰ and Astana Snowmelt Water (ASMW) δ ^18^O = −12.67 ± 0.15‰, δ^2^H = −82.1 ± 0.6‰. The analyses were performed with 2 preparatory and 6–8 measured injections in high precision mode for each sample. The four last injections with minimal standard deviations were usually used for normalization. In case of large deviations (exceeding the instrument's uncertainties) between injections for a particular sample, the measurements were repeated to obtain the reproducible result. The post-processing was done in LRG post analysis software (Version 4.5.06) using bracketing normalization and spectral contamination screening.

## Limitations

The present dataset was primarily exploratory in nature based mostly on wide-range sampling. The generated Local Meteoric Water Line for Astana is based on approximately two years of event-based collection. Monthly cumulative values included heatwave/drought years with low amount of warm season precipitation (2021–2022). There were also initial problems with Palmex precipitation sampler with precipitation undercatch, absence of snow tube and siphon (winter) inlet (resolved in autumn 2023) resulting in very low amounts of the sample collected. If there was a sufficient sample amount for analysis it was processed and incorporated into the dataset. In such a case the note was added to the comment's column. The undercatch was resolved by upgrading from 135 mm diameter funnel to 235 mm diameter. The sampling location in Astana at Nazarbayev University is the site of Global Network of Isotopes in Precipitation (GNIP). The sample analysis quality was assessed through completion of a round-robin test supplied by International Atomic Energy Agency's Isotope Hydrology Section as outlined in [13].

## Ethics Statement

The current work does not involve human subjects, animal experiments, or any data collected from social media platforms. The manuscript and presented data are the author's own original work that has not been published previously elsewhere.

## CRediT authorship contribution statement

**Vadim Yapiyev:** Conceptualization, Data curation, Methodology, Formal analysis, Validation, Writing – original draft, Writing – review & editing, Project administration, Funding acquisition, Resources, Visualization. **Nurlan Ongdas:** Data curation, Formal analysis, Investigation, Validation, Writing – original draft, Writing – review & editing, Funding acquisition, Visualization. **Sylvia Pinkerneil:** Investigation, Writing – review & editing. **Kanat Samarkhanov:** Investigation. **Arman Kabdeshev:** Investigation. **Yergali Karakulov:** Investigation. **Murat Muzdybaev:** Investigation. **Aksholpan Atalikhova:** Project administration. **Catalin Stefan:** Resources, Funding acquisition. **Jay Sagin:** Resources, Funding acquisition. **Milovan Fustic:** Resources, Funding acquisition.

## Data Availability

The exploratory dataset of isotopic composition of different water sources across Kazakhstan. (Original data) (Mendeley Data)

## References

[1] Yapiyev V., Ongdas N., Pinkerneil S., Samarkanov K., Kabdeshev A., Karakulov Y., Muzdybaev M., Atalikhova A., Stefan C., Sagin J., Fustic M. (2024). The exploratory dataset of isotopic composition of different water sources across Kazakhstan. Mendeley Data.

[2] Yapiyev V., Skrzypek G., Verhoef A., Macdonald D., Sagintayev Z. (2020). Between boreal Siberia and arid Central Asia – stable isotope hydrology and water budget of Burabay National Nature Park ecotone (Northern Kazakhstan). J. Hydrol. Reg. Stud..

[3] Vystavna Y., Harjung A., Monteiro L.R., Matiatos I., Wassenaar L.I. (2021). Stable isotopes in global lakes integrate catchment and climatic controls on evaporation. Nat. Commun..

[4] Jasechko S., Birks S.J., Gleeson T., Wada Y., Fawcett P.J., Sharp Z.D., McDonnell J.J., Welker J.M. (2014). The pronounced seasonality of global groundwater recharge. Water Resour. Res..

[5] Jasechko S., Wassenaar L.I., Mayer B. (2017). Isotopic evidence for widespread cold-season-biased groundwater recharge and young streamflow across central Canada. Hydrol. Process..

[6] Jasechko S., Lechler A., Pausata F.S.R., Fawcett P.J., Gleeson T., Cendon D.I., Galewsky J., LeGrande A.N., Risi C., Sharp Z.D., Welker J.M., Werner M., Yoshimura K. (2015). Late-glacial to late-Holocene shifts in global precipitation δ18O. Clim. Past.

[7] Crawford J., Hughes C.E., Lykoudis S. (2014). Alternative least squares methods for determining the meteoric water line, demonstrated using GNIP data. J. Hydrol..

[8] Wu H., Wu J., Song F., Abuduwaili J., Saparov A.S., Chen X., Shen B. (2019). Spatial distribution and controlling factors of surface water stable isotope values (δ18O and δ2H) across Kazakhstan, Central Asia. Sci. Total Environ..

[9] Shen B., Wu J., Zhan S., Jin M., Saparov A.S., Abuduwaili J. (2021).

[10] Kozhagulova A., Yapiyev V., Karabayanova L., Dillinger A., Zavaley V., Kalitova A., Bayramov E., Holbrook J., Grasby S.E., Fustic M. (2023). Geological controls on the geothermal system and hydrogeochemistry of the deep low-salinity Upper Cretaceous aquifers in the Zharkent (eastern Ily) Basin, south-eastern Kazakhstan. Front. Earth Sci..

[11] Craig H. (1961). Isotopic variations in meteoric waters. Science.

[12] van Geldern R., Barth J.A.C. (2012). Optimization of instrument setup and post-run corrections for oxygen and hydrogen stable isotope measurements of water by isotope ratio infrared spectroscopy (IRIS). Limnol. Oceanogr. Methods.

[13] Wassenaar L., Terzer-Wassmuth S., Douence C. (2021). Progress and challenges in dual- and triple-isotope (δ 18 O, δ 2 H, Δ 17 O) analyses of environmental waters: an international assessment of laboratory performance. Rapid Commun. Mass Spectrom..

